# OASes and STING: Adaptive Evolution in Concert

**DOI:** 10.1093/gbe/evv046

**Published:** 2015-03-09

**Authors:** Alessandra Mozzi, Chiara Pontremoli, Diego Forni, Mario Clerici, Uberto Pozzoli, Nereo Bresolin, Rachele Cagliani, Manuela Sironi

**Affiliations:** ^1^Bioinformatics, Scientific Institute IRCCS E.MEDEA, Bosisio Parini, Italy; ^2^Department of Physiopathology and Transplantation, University of Milan, Italy; ^3^Don C. Gnocchi Foundation ONLUS, IRCCS, Milan, Italy; ^4^Department of Physiopathology and Transplantation, Dino Ferrari Centre, University of Milan, Fondazione Ca’ Granda IRCCS Ospedale Maggiore Policlinico, Milan, Italy

**Keywords:** OAS, cGAS, STING, RNase L, positive selection

## Abstract

OAS (2′–5′-oligoadenylate synthases) proteins and cyclic GMP–AMP synthase (cGAS, gene symbol: *MB21D1*) patrol the cytoplasm for the presence of foreign nucleic acids. Upon binding to double-stranded RNA or double-stranded DNA, OAS proteins and cGAS produce nucleotide second messengers to activate RNase L and STING (stimulator of interferon genes, gene symbol: *TMEM173*), respectively; this leads to the initiation of antiviral responses. We analyzed the evolutionary history of the *MB21D1–TMEM173* and *OAS–RNASEL* axes in primates and bats and found evidence of widespread positive selection in both orders. In *TMEM173*, residue 230, a major determinant of response to natural ligands and to mimetic drugs (e.g., DMXAA), was positively selected in Primates and Chiroptera. In both orders, selection also targeted an α-helix/loop element in RNase L that modulates the enzyme preference for single-stranded RNA versus stem loops. Analysis of positively selected sites in *OAS1*, *OAS2*, and *MB21D1* revealed parallel evolution, with the corresponding residues being selected in different genes. As this cannot result from gene conversion, these data suggest that selective pressure acting on *OAS* and *MB21D1* genes is related to nucleic acid recognition and to the specific mechanism of enzyme activation, which requires a conformational change. Finally, a population genetics-phylogenetics analysis in humans, chimpanzees, and gorillas detected several positively selected sites in most genes. Data herein shed light into species-specific differences in infection susceptibility and in response to synthetic compounds, with relevance for the design of synthetic compounds as vaccine adjuvants.

## Introduction

The innate immune system recognizes invading infectious agents through an array of so-called pattern-recognition receptors (PRRs). These molecules detect pathogen-associated molecular patterns (PAMPs) and initiate a downstream signaling cascade that ultimately triggers antiviral/antimicrobial programs. PRRs belong to diverse molecular families including Toll-like receptors (TLRs), Nod-like receptors, RIG-I-like receptors, and AIM2-like receptors (ALRs).

Recently, a cytosolic cyclic GMP–AMP synthase (cGAS, official gene symbol: *MB21D1*) was found to act as an antiviral DNA sensor (Sun et al. 2013). Upon binding to DNA, cGAS catalyzes the synthesis of cyclic GMP–AMP (cGAMP), which functions as a second messenger and binds the stimulator of interferon genes (STING, official gene symbol: *TMEM173*). STING, which is located in the endoplasmic reticulum (ER), can also sense cyclic dinucleotides of prokaryotic origin and is targeted by different viruses, including hepatitis C virus (HCV) and Dengue virus.

cGAS can detect a wide range of viruses and shares structural and functional features with OAS1 (2′–5′-oligoadenylate synthase 1). Although they are not phylogenetically related, OAS1 and cGAS display similar structural fold and activation mechanisms, and both enzymes produce atypical nucleotide second messengers. In fact, OAS1 and its paralogs, OAS2 and OAS3, have long been known to bind viral double-stranded RNA (dsRNA) and to catalyze the synthesis of 2′–5′ oligoadenylates, which specifically activate the latent form of RNase L (Hovanessian et al. 1977; Kerr and Brown 1978). Inhibition of viral propagation is eventually achieved by RNase L through RNA degradation and induction of apoptosis.

Based on their similarities, cGAS and OAS proteins may be considered as a novel family of PRRs (Civril et al. 2013; Kranzusch et al. 2013), although they impinge on different effector molecules.

Because of their direct role in PAMP recognition, PRRs and their downstream effectors are constantly involved in genetic conflicts with pathogens and, as a consequence, are commonly targeted by positive selection (Wlasiuk and Nachman 2010; Areal et al. 2011; Cagliani, Forni, Tresoldi, et al. 2014; Cagliani, Forni, Biasin, et al. 2014; Tenthorey et al. 2014). RNase L, for example, evolved adaptively in Primates, with most positively selected sites located in protein domains that directly contact the viral genetic material (Jin et al. 2012). This is in line with the host–pathogen arms race scenario, whereby protein regions directly involved in the recognition and binding of pathogen-derived components should evolve under the strongest selective pressure. This implies that species-specific differences in the function of PRRs or of their downstream effectors may be common. In the case of TLRs, for instance, the same receptor in distinct species may recognize different ligands or the same ligand with different affinity (Werling et al. 2009). Therefore, studying the pattern of interspecies evolution may provide valuable information on the differential susceptibility to infection within and among species. Primates, for example, show marked differences in the susceptibility and severity of several viral infections including those caused by HIV/SIV (Simian Immunodeficiency Virus), HCV, HBV, and Varicella-zoster virus (Varki 2000; Willer et al. 2012). Moreover, several emerging and re-emerging viral diseases affecting humans originate through the zoonotic transmission from a reservoir animal host (Jones et al. 2008). Recent examples of pathogen spillover events include the Ebola and Middle East respiratory syndrome (MERS-CoV) viruses: Both originated in bats and subsequently spread to humans either directly or through an intermediate host (Wang et al. 2011; Cotten et al. 2013). Indeed, bats (Chiroptera) have long been known to harbor and disseminate a wide range of viruses that are highly pathogenic for humans. In addition to Ebola virus and MERS-CoV, notable examples include henipaviruses (e.g., Nipah and Hendra viruses), which cause a high fatality rate in humans and other mammals, hepaciviruses, influenza A viruses, as well as a range of paramyxoviruses (Calisher et al. 2006; Drexler et al. 2012; Tong et al. 2012; Quan et al. 2013). With the exception of lyssavirus (e.g., rabies virus), bats are symptomless carriers of these human viral pathogens (Field et al. 1999). On the one hand, the observation whereby several Chiroptera families harbor a range of viral species suggests that bats have been coevolving with viruses for a long time and have adapted to high viral exposure. On the other hand, the wide variety of viral families hosted by bats indicates that adaptation most likely involved genes with a role in immune response, rather than molecules acting as incidental viral receptors (as different viruses use distinct strategies to invade the host). Thus, innate immunity genes that are devoted to antiviral response represent excellent candidates as adaptive selection targets in Chiroptera. A recent comparison of two bat genomes (*Pteropus alecto* and *Myotis davidii*) reported adaptive evolution at such genes, including *TLR7* and *TBK1*, this latter encoding an interactor of STING (Zhang, Cowled, et al. 2013). Nonetheless, fast evolutionary rates at immune response loci are a common feature of mammalian genomes and surely do not represent a bat-specific trait (Barreiro and Quintana-Murci 2010; Zhang, Cowled, et al. 2013).

Also, an unexpected finding emerged from the analysis of the two bat genomes, as both species were found to have lost the entire cluster of *ALR* genes (Zhang, Cowled, et al. 2013). Overall, as noted elsewhere (Wynne and Wang 2013), the adaptive strategies underlying bat ability to asymptomatically maintain viruses remain elusive. Possibly, detailed analyses of specific antiviral systems may help address this issue. Starting from this premise, we analyzed the evolutionary history of the *OAS–RNASEL* and *MB21D1–TMEM173* axes in primates and bats.

## Materials and Methods

### Gorilla Sample and Sequencing

The genomic DNA of one *Gorilla gorilla* was obtained from the European Collection of Cell Cultures (ECACC). *MB21D1* exons 3 and 5 were polymerase chain reaction (PCR)-amplified from genomic DNA and directly sequenced using primers 5′-GCCTGAACATATAACATTAAC-3′ (exon 3) and 5′-AGGGTGACTCTAGTTCTTAGA-3′ (exon 5) as forward and 5′-TTATTTCCCCTGTATTTCCAG-3′ (exon 3) and 5′-GCTATGAGATGCCTAAAATCC-3′ (exon 5) as reverse. PCR products were treated with ExoSAP-IT (USB Corporation, Cleveland, OH), directly sequenced on both strands with a Big Dye Terminator sequencing Kit (v3.1 Applied Biosystems), and run on an Applied Biosystems ABI 3130 XL Genetic Analyzer (Life Technologies). Sequences were assembled using AutoAssembler version 1.4.0 (Applied Biosystems), and manually inspected. The obtained sequences have been submitted to the National Center for Biotechnology Information (NCBI) database.

### Evolutionary Analyses in Primates and Bats

Primate and bat sequences were retrieved from the NCBI database (http://www.ncbi.nlm.nih.gov, last accessed October 31, 2014). The tree shrew and the horse sequences were also included in primate and bat alignments, respectively. A list of species is reported in supplementary table S2, Supplementary Material online. DNA alignments were performed using the RevTrans 2.0 utility (http://www.cbs.dtu.dk/services/RevTrans/, last accessed October 31, 2014) (Wernersson and Pedersen 2003), which uses the protein sequence alignment as a scaffold to construct the corresponding DNA multiple alignment. This latter was checked and edited by TrimAl to remove alignment uncertainties (http://phylemon.bioinfo.cipf.es/utilities.html, last accessed October 31, 2014) (Capella-Gutierrez et al. 2009). Gene trees were generated by maximum likelihood using the program phyML (Guindon et al. 2009).

Positive selection was detected using PAML (Phylogenetic Analysis by Maximum Likelihood) analyses (Yang 2007). The site models implemented in PAML were developed to detect positive selection affecting only a few amino acid residues in a protein: Positive selection is characterized by a nonsynonymous substitution/synonymous substitution rate (d*N*/d*S*, also referred to as ω) ratio > 1. To detect selection, site models that allow (M2a and M8) or disallow (M1a and M7) a class of sites to evolve with ω > 1 were fitted to the data using the F3x4 model (codon frequencies estimated from the nucleotide frequencies in the data at each codon site) and the F61 model (frequencies of each of the 61 nonstop codons estimated from the data).

Positively selected sites were identified using two different methods: The Bayes Empirical Bayes (BEB) analysis (with a cutoff of 0.90), which calculates the posterior probability that each codon is from the site class of positive selection (under model M8) (Anisimova et al. 2002), and the Mixed Effects Model of Evolution (MEME) (with the default cutoff of 0.1) (Murrell et al. 2012), which allows the distribution of ω to vary from site to site and from branch to branch at a site. Only sites detected using both methods were considered positively selected.

To explore also possible variations in selective pressure among different lineages, we applied the free-ratio models implemented in the PAML package: The M0 model assumes all branches to have the same ω, whereas M1 allows each branch to have its own ω (Yang 1997). The models are compared through likelihood-ratio tests (LRT) (degree of freedom = total number of branches − 1). In order to identify specific branches with a proportion of sites evolving with ω > 1, we used branch site-random effects likelihood (BS-REL) (Kosakovsky Pond et al. 2011). This method implements branch-site models that simultaneously allow ω variation across branches and sites. BS-REL requires no prior knowledge about which lineages are more likely to have experienced episodic diversifying selection. Branches identified using this approach were cross-validated with the branch-site LRT from PAML (the so-called modified model A and model MA1, “test 2”) (Zhang et al. 2005). A false discovery rate (FDR) correction was applied to account for multiple hypothesis testing (i.e., we corrected for the number of tested lineages), as suggested (Anisimova and Yang 2007). MEME and BEB analysis from MA (with a cutoff of 0.90) were used to identify sites that evolve under positive selection on specific lineages (Zhang et al. 2005). 

Genetic algorithm recombination detection (GARD) (Kosakovsky Pond et al. 2006), single-likelihood ancestor counting (SLAC) (Kosakovsky Pond and Frost 2005), MEME (Murrell et al. 2012), and BS-REL analyses were performed through the DataMonkey server (http://www.datamonkey.org, last accessed October 31, 2014) (Delport et al. 2010) or run locally (through HyPhy).

### Population Genetics–Phylogenetics Analysis

Data from the Pilot 1 phase of the 1000 Genomes Project (1000G) were retrieved from the dedicated website (http://www.1000genomes.org/, last accessed October 31, 2014) (1000 Genomes Project Consortium et al. 2010). Single nucleotide polymorphism (SNP) genotype information for 25 unrelated chimpanzees and 27 unrelated gorillas were retrieved from Prado-Martinez et al. (2013). Coding sequence information was obtained for each gene and the ancestral sequence was reconstructed by parsimony from the human, chimpanzee, orangutan, and macaque sequences. Analyses were performed with gammaMap (Wilson et al. 2011).

For gammaMap analysis, we assumed θ (neutral mutation rate per site), *k* (transitions/transversions ratio), and *T* (branch length) to vary among genes following log-normal distributions. For each gene, we set the neutral frequencies of non-STOP codons (1/61) and the probability that adjacent codons share the same selection coefficient (*P* = 0.02). For selection coefficients, we considered a uniform Dirichlet distribution with the same prior weight for each selection class. For each gene, we run 100,000 iterations with thinning interval of ten iterations.

To be conservative, we declared a codon to be targeted by positive selection when the cumulative posterior probability of γ ≥ 1 was greater than 0.75, as suggested (Quach et al. 2013).

### Three-Dimensional Structure Analysis

Protein three-dimensional (3D) structures for human OAS1 (PDB code: 4IG8) (Donovan et al. 2013), cGAS (PDB codes: 4O67 and 4KM5) (Kranzusch et al. 2013; Zhang et al. 2014), RNase L (PDB code: 4OAV) (Han et al. 2014), and STING (PDB codes: 4LOH, 4QXP, and 4KSY) (Gao, Ascano, Zillinger, et al. 2013; Zhang, Shi, et al. 2013; Gao et al. 2014) were derived from the Protein Data Bank (PDB) (http://www.pdb.org, last accessed October 31, 2014); the human OAS2 model was obtained from the Protein Model Portal (code: P29728). Structure superimposition and sites mapping were performed using PyMOL (The PyMOL Molecular Graphics System, Version 1.5.0.2 Schrödinger, LLC).

## Results

### Adaptive Evolution in Primates

We analyzed the evolutionary history of *OAS* genes (including the enzymatically inactive *OASL*), *MB21D1*, and *TMEM173* in Primates (supplementary table S1, Supplementary Material online). Although Jin et al. (2012) previously described adaptive evolution of *RNASEL* in Primates, we included the gene to allow comparison with bats and mapping of selected sites on the 3D structure, which has recently been solved (see below). Coding sequences for available primate species were retrieved from public databases; the tree shrew sequence was also included as an outgroup (supplementary table S2, Supplementary Material online). Direct sequencing of *G. gorilla* DNA was used to fill-in gaps in the coding sequence of *MB21D1*.

DNA alignments were generated using RevTrans (Wernersson and Pedersen 2003) and screened for the presence of recombination using GARD (Kosakovsky Pond et al. 2006). No breakpoint was detected for any gene.

**Fig. 1.—:**
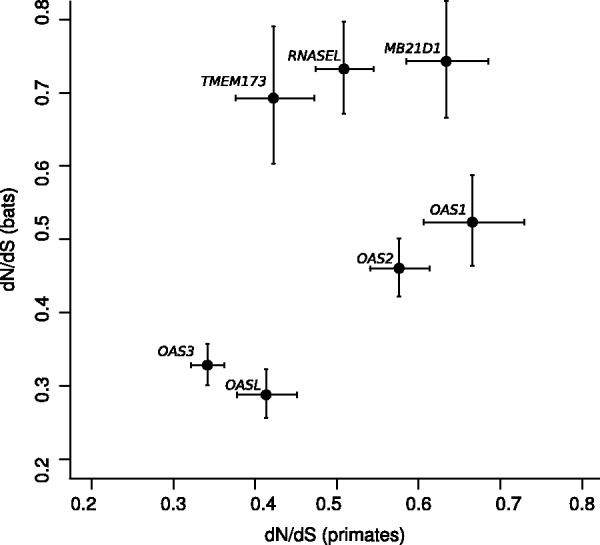
Plot of d*N*/d*S* values (with 95% confidence intervals) calculated for *OAS* family genes, *MB21D1*, *RNASEL*, and *TMEM173* in primates and bats.

The average nonsynonymous/synonymous substitution rate ratio (d*N*/d*S*, also referred to as ω) was calculated using the SLAC method (Kosakovsky Pond and Frost 2005). In analogy to most mammalian genes (Lindblad-Toh et al. 2011), d*N*/d*S* was always lower than 1 (fig. 1), indicating purifying selection as the major force shaping diversity at these genes in Primates. This finding does not exclude that localized positive selection acts on specific sites or domains. To test this possibility, we applied LRT implemented in the “codeml” program (Yang 1997, 2007).

Under different codon frequency models, two neutral models (M1a and M7) were rejected in favor of the positive selection models (M2a and M8) for *OAS1*, *OAS2*, *MB21D1*, *TMEM173*, and *RNASEL* (table 1, supplementary table S3, Supplementary Material online). No evidence of positive selection was detected for *OAS3* and *OASL*.

**Table 1:** Likelihood Ratio Test Statistics for Models of Variable Selective Pressure among Sites (codon frequency model:F3x4)

Gene/Selection Model	*N* Species	−2ΔLn *L*	*P* Value	% of Sites (average d*N*/d*S*)
*OAS1*				
M1a versus M2a				
Primates	17	56.91	4.40 × 10^−13^	20.0 (2.9)
Chiroptera	7	32.49	8.77 × 10^−8^	7.0 (6.4)
M7 versus M8				
Primates	17	62.14	3.22 × 10^−14^	23.6 (2.7)
Chiroptera	7	33.10	6.50 × 10^−8^	7.0 (6.3)
*OAS2*				
M1a versus M2a				
Primates	16	63.19	1.89 × 10^−14^	7.9 (3.3)
M7 versus M8				
Primates	16	89.44	3.78 × 10^−20^	12.6 (2.7)
*MB21D1*				
M1a versus M2a				
Primates	16	37.05	8.99 × 10^−9^	8.9 (3.2)
Chiroptera	6	12.49	0.002	35.1 (2.0)
M7 versus M8				
Primates	16	45.89	1.08 × 10^−10^	11.4 (2.9)
Chiroptera	6	13.10	0.001	35.0 (2.0)
*RNASEL*				
M1a versus M2a				
Primates	21	41.32	1.06 × 10^−9^	6.0 (3.2)
Chiroptera	7	44.19	2.53 × 10^−10^	9.8 (4.2)
M7 versus M8				
Primates	21	56.06	6.70 × 10^−13^	9.5 (2.6)
Chiroptera	7	44.13	2.61 × 10^−10^	10.3 (4.1)
*TMEM173*				
M1a versus M2a				
Primates	17	6.62	0.04	3.3 (2.5)
Chiroptera	7	32.91	7.14 × 10^−8^	16.9 (4.1)
M7 versus M8				
Primates	17	7.44	0.02	11.3 (1.8)
Chiroptera	7	33.32	5.81 × 10^−8^	16.8 (4.1)

Note.—M1a is a nearly neutral model that assumes one ω class between 0 and 1 and one class with ω = 1; M2a (positive selection model) is the same as M1a plus an extra class of ω > 1; M7 (null model) assumes that 0 < ω < 1 is beta distributed among sites in ten classes; M8 (selection model) has an extra class with ω ≥ 1; 2ΔLn *L*, twice the difference of the natural logs of the maximum likelihood of the models being compared; *P* Value, *P* value of rejecting the neutral models (M1a or M7) in favor of the positive selection model (M2a or M8); % of sites (average d*N*/d*S*), estimated percentage of sites evolving under positive selection by M8 and M2a (d*N*/d*S* for these codons).

**Fig. 2.—:**
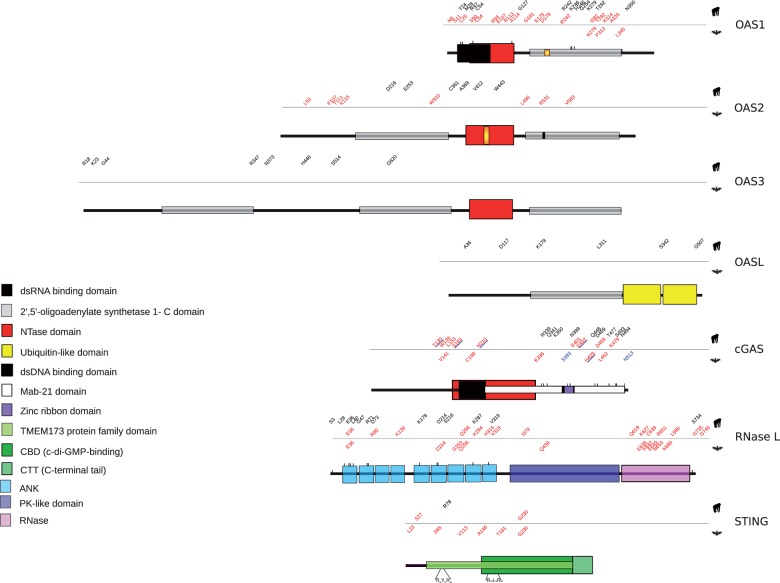
Schematic representation of the domain structure of OASs family members, cGAS, RNase L, and STING. Domains are color-coded as reported in the legend (left). The position of positively selected sites is shown and color-coded as follows: Red, positively selected sites in the primate or bat phylogenies; blue, lineage-specific positively selected sites; black, positively selected sites in the human, chimpanzee, or gorilla lineages.

We next applied the BEB analysis (Anisimova et al. 2002; Yang et al. 2005) and the MEME (Murrell et al. 2012) to identify specific sites targeted by positive selection in these genes; only sites detected using both methods were considered (fig. 2).

**Fig. 3.—:**
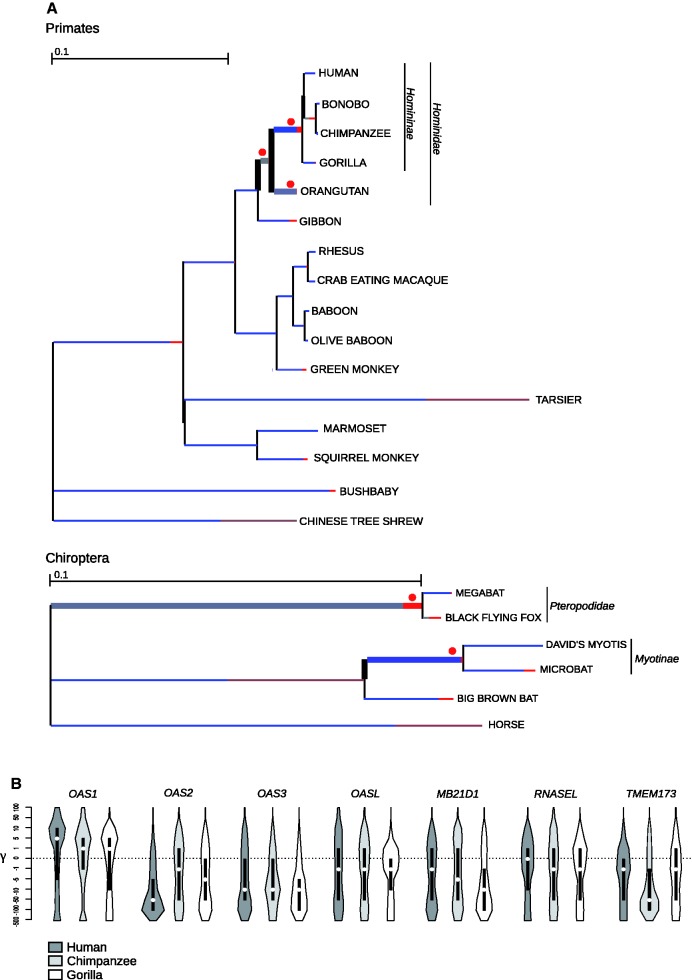
(*A*) Branch-site analysis of positive selection for *MB21D1* gene (cGAS) in Primates and Chiroptera. Branch lengths are scaled to the expected number of substitutions per nucleotide, and branch colors indicate the strength of selection (ω). Red, positive selection (ω > 5); blue, purifying selection (ω = 0); gray, neutral evolution (ω = 1). The proportion of each color represents the fraction of the sequence undergoing the corresponding positive class of selection. Thick branches indicate statistical support for evolution under episodic diversifying selection as determined by BS-REL. Red dots denote branches that were also detected to be under selection using the PAML branch-site models. (*B*) Violin plot of selection coefficients (median, white dot; interquartile range, black bar). Selection coefficients (γ) are classified as strongly beneficial (100, 50), moderately beneficial (10, 5), weakly beneficial (1), neutral (0), weakly deleterious (−1), moderately deleterious (−5, −10), strongly deleterious (−50, −100), and inviable (−500).

Finally, we extended our analysis to explore possible variations in selective pressure across primate lineages. To this aim, we tested whether models that allow d*N*/d*S* to vary along branches had significant better fit than models that assume one same d*N*/d*S* across the entire phylogeny (Yang and Nielsen 1998). This hypothesis was verified for *OAS1, MB21D1,* and *RNASEL* (supplementary table S4, Supplementary Material online). We thus used the BS-REL method (Kosakovsky Pond et al. 2011) to analyze selection along specific lineages. BS-REL identified two branches for *OAS1*, three for *MB21D1,* and two for *RNASEL* (fig. 2 and supplementary table S4, Supplementary Material online). These were cross-validated using codeml (branch-site LRT models) (Zhang et al. 2005), with application of FDR correction, as suggested (Anisimova and Yang 2007). The analysis did not confirm the O*AS1* branches detected by BS-REL. Conversely for the *MD21D1* gene all the three branches were validated and positively selected sites were identified for the Hominidae and Homininae lineages (fig. 3*A* and supplementary table S4, Supplementary Material online). Finally, for *RNASEL* only the Tibetan macaque branch was confirmed but no positively selected sites were found (supplementary fig. S1, Supplementary Material online).

### Positive Selection in Primate Lineages

To gain insight into the more recent selective events in Primates, we applied a population genetics–phylogenetics approach to study the evolution of *OAS* genes, *MB21D1*, *TMEM173*, and *RNASEL* in the human, chimpanzee, and gorilla lineages. Specifically, we applied gammaMap (Wilson et al. 2011) that jointly uses intraspecies variation and interspecific diversity to estimate the distribution of selection coefficients (γ) along coding regions.

For humans, we exploited data from the 1000 Genomes Pilot Project (1000G) for Europeans (CEU), Yoruba (YRI), and Chinese plus Japanese (CHBJPT) (1000 Genomes Project Consortium et al. 2010). For chimpanzees and gorillas, we used SNP information from 25 and 27 individuals, respectively (Prado-Martinez et al. 2013).

We also used gammaMap to identify specific codons evolving under positive selection (defined as those having a cumulative probability greater than 0.75 of γ ≥ 1) in each lineage.

Results indicated that in the three species, most genes evolved under different degrees of purifying selection, with the exclusion of *OAS1*, which showed a preponderance of sites with γ values in the 5–10 range (i.e., moderately beneficial) (fig. 3*B*). The distribution of γ values for *OAS1* was similar in humans, chimpanzees, and gorillas. In general, γ distributions at the seven genes were comparable in the three species with the exclusion of *OAS2* in humans and *TMEM173* in chimpanzees, which showed stronger constraint compared with the other two species (fig. 3*B*).

We also detected sites targeted by positive selection at most genes. In *OAS1,* some positively selected sites were shared by two or even three (codon 54) species (table 2). Interestingly, most of these *OAS1* sites were previously shown to define two major haplotype clades that segregate in chimpanzees and are maintained by long-standing balancing selection (Ferguson et al. 2012) (table 2). The most recent common ancestor of the two haplotype clades was estimated to predate the human/chimpanzee/gorilla split (Ferguson et al. 2012). Because the ancient-balanced haplotypes carry several coding variants and because some of these were also detected as positively selected sites in the BEB analyses (table 2), we reran the PAML site models after masking these sites in the human, chimpanzee, and gorilla sequences. Fully significant results were obtained in all LRT (data not shown), indicating that the positive selection signal at *OAS1* is not merely accounted for by the long-standing balancing selection event in hominids.

**Table 2:** Positively Selected Sites in the Human, Chimpanzee, and Gorilla Lineages

Gene	Lineage	Codon	Ancestral AA	Derived AA	Pr^a^	DAF^b^	Other Methods^c^
*OAS1*							
	Human	54^d^	Arg	Cys	0.868	1	MEME–BEB
		127	Asp	Gly	0.928	1	
		279^d^	Glu	Lys	0.885	1	BEB
		292^d^	Arg	Thr	0.976	1	MEME–BEB
		350	Asp	Asn	0.963	1	
	Chimpanzee	24^d^	Thr	Lys	0.988	1	
		28^d^	Met	Lys	0.990	1	BEB
		47^d^	Arg	Gln	0.982	1	
		54^d^	Arg	His	0.972	1	MEME–BEB
		242^d^	Arg	Gln	0.834	0.62	MEME–BEB
		246^d^	Lys	Glu	0.845	0.60	BEB
		248^d^	His	Asp	0.847	0.60	BEB
		254^d^	Gly	Glu	0.842	0.61	
		292^d^	Arg	Thr/Glu	0.941	0.62/0.38	MEME–BEB
		355	Trp	Stop^e^	0.775	0.62	
	Gorilla	54^d^	Arg	Cys	0.818	1	MEME–BEB
		69	Thr	Ala	0.823	1	BEB
		127	Asp	Gly	0.846	1	
		175	Glu	Lys	0.940	1	MEME–BEB
		179	Asp	Tyr	0.936	1	MEME–BEB
		242^d^	Arg	Gln	0.807	1	MEME–BEB
		279^d^	Glu	Lys	0.850	1	BEB
*OAS2*							
	Chimpanzee	216	Asp	Asn	0.834	1	
		253	Glu	Lys	0.828	1	BEB
	Gorilla	361	Cys	Phe	0.869	1	BEB
		369	Ala	Thr	0.867	1	
		412	Val	Ile	0.799	1	
		443	Trp	Ser	0.767	1	MEME–BEB
*OAS3*							
	Human	446	Arg	His	0.828	1	
		514	Gly	Ser	0.860	1	MEME–BEB
		620	Arg	Gly	0.837	1	
	Chimpanzee	347	Arg	Cys	0.794	1	MEME–BEB
		373	Asn	Ser	0.773	0.96	MEME–BEB
	Gorilla	18	Arg	Ser	0.870	1	
		23	Lys	Thr	0.873	1	
		44	Gly	Ala	0.818	1	
*OASL*							
	Human	179	Glu	Lys	0.857	1	
		311	His	Leu	0.857	1	
		432	Pro	Ser	0.856	1	
	Chimpanzee	36	Ala	Thr	0.799	1	
		117	Asp	Asn	0.759	1	
		507	Gly	Arg	0.839	1	
*MB21D1*							
	Human	339	Pro	Arg	0.909	1	MEME
		341	Lys	Gln	0.910	1	
	Chimpanzee	399	Asn	Ile	0.854	1	
		448	Gln	Glu	0.910	1	
		477	Thr	Ile	0.912	1	
		494	Asn	Asp	0.894	1	
	Gorilla	339	Pro	Arg	0.931	1	MEME
		341	Lys	Gln	0.932	1	
		350	Lys	Arg	0.900	1	BEB
		459	Asp	Gln	0.898	1	BEB
		493	Ser	Arg	0.774	1	
*RNASEL*							
	Human	3	Thr	Ser	0.823	1	
		178	Glu	Lys	0.816	1	BEB
		214	His	Asp	0.928	1	BEB
		216	Arg	Ser	0.928	1	MEME
		287	Glu	Lys	0.802	1	MEME
		319	Phe	Val	0.781	1	MEME–BEB
	Chimpanzee	28	Leu	Ser	0.987	1	
		36	Glu	Gly	0.997	1	MEME–BEB
		40	Leu	Gln	0.998	1	MEME
		47	Gly	Asp	0.995	1	BEB
		71	Arg	Lys	0.985	1	MEME
		73	Asp	Glu	0.982	1	MEME
		734	Ser	Cys	0.806	1	
*TMEM173*							
	Chimpanzee	78	Arg	Trp	0.759	1	

^a^Posterior probability of γ ≥ 1 as detected by gammaMap.

^b^Derived allele frequency.

^c^Other methods that identified the same codon as positively selected.

^d^Site described as long-term balancing selection target (see text).

^e^To perform GammaMap analyses, the STOP codon was substituted with a different codon.

Two positively selected sites shared between humans and gorillas were also detected at *MB21D1* (fig. 2, table 2).

### Positive Selection in Chiroptera

As a comparison to Primates and given the role of these mammals as virus reservoirs, we analyzed the evolution of genes in the *OAS–RNASEL* and *MB21D1–TMEM173* axes in bats. Specifically, we obtained coding sequences for at least six bat species from public databases and we included the horse sequence as an outgroup (supplementary table S2, Supplementary Material online). As for Primates, the average d*N*/d*S* substitution rate ratio, calculated using SLAC (Kosakovsky Pond and Frost 2005), was in all cases lower than 1. Comparison with Primates revealed a good correspondence in d*N*/d*S* across the seven genes, except for*TMEM173* and *RNASEL*, which showed comparatively higher values in bats than in primates (fig. 1).

Application of the codeml site models indicated the action of positive selection for *OAS1, MB21D1, TMEM173,* and *RNASEL* (table 1). BEB and MEME analyses identified positively selected sites in the four genes (fig. 2).

Variations in selective pressure among bat lineages were detected for *OAS1* and *MB21D1* (supplementary table S4, Supplementary Material online); BS-REL identified the horse branch for *OAS1,* the Myotinae and Pteropodidae branches for both genes (fig. 3*A* and supplementary fig. S1*A* and table S4, Supplementary Material online). With the exception of Myotinae branch for *OAS1*, c*odeml* analysis with FDR correction confirmed all branches, but BEB and MEME analyses detected lineage-specific positively selected sites in *MB21D1* only (fig. 2 and supplementary table S4, Supplementary Material online).

### Parallel Evolution at OAS1, OAS2, and cGAS

**Fig. 4.—:**
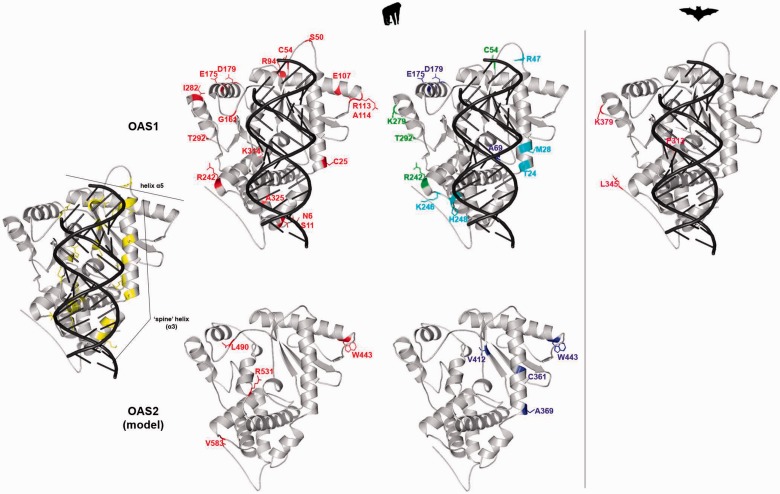
Positively selected sites mapped onto the human OAS1 (PDB code: 4IG8) and OAS2 (model, code: P29728) structures. Color codes are as follows: Yellow, residues involved in dsRNA binding mapped onto OAS1; red, positively selected sites in the whole primate or bat phylogenies; cyan, positively selected sites in the chimpanzee lineage; blue, positively selected sites in the gorilla lineage; green, positively selected sites in more than one lineage among human, chimpanzee, and gorilla.

We identified several selected sites in *OAS1*; as previously noted (Ferguson et al. 2012), some residues (e.g., T24, M28, R47, C54) defining the two haplotypes maintained by long-standing balancing selection are located along the so-called “spine” helix (helix α3) (fig. 4). This helix is central for human OAS1 function: It acts as a platform for nucleic acid binding and undergoes a dsRNA-induced structural switch (Donovan et al. 2013; Hornung et al. 2014). Additional sites (S11 and S50) positively selected in the whole primate phylogeny are located on this helix (fig. 4).

Comparison of the OAS1 3D structures with the OAS2 model predicted that OAS1 residue C25 corresponds to A369 in OAS2, which is positively selected in the gorilla lineage. Also, E107–R113–A114 and W443, positively selected in OAS1 and OAS2, respectively, lie at the C-terminus of helix α5, an element that is also subjected to a structural shift after dsRNA binding (Donovan et al. 2013). Finally, OAS2 residue 490 is predicted to be spatially close to residue 161 in OAS1; both sites are positively selected in the primate phylogeny (fig. 4).

**Fig. 5.—:**
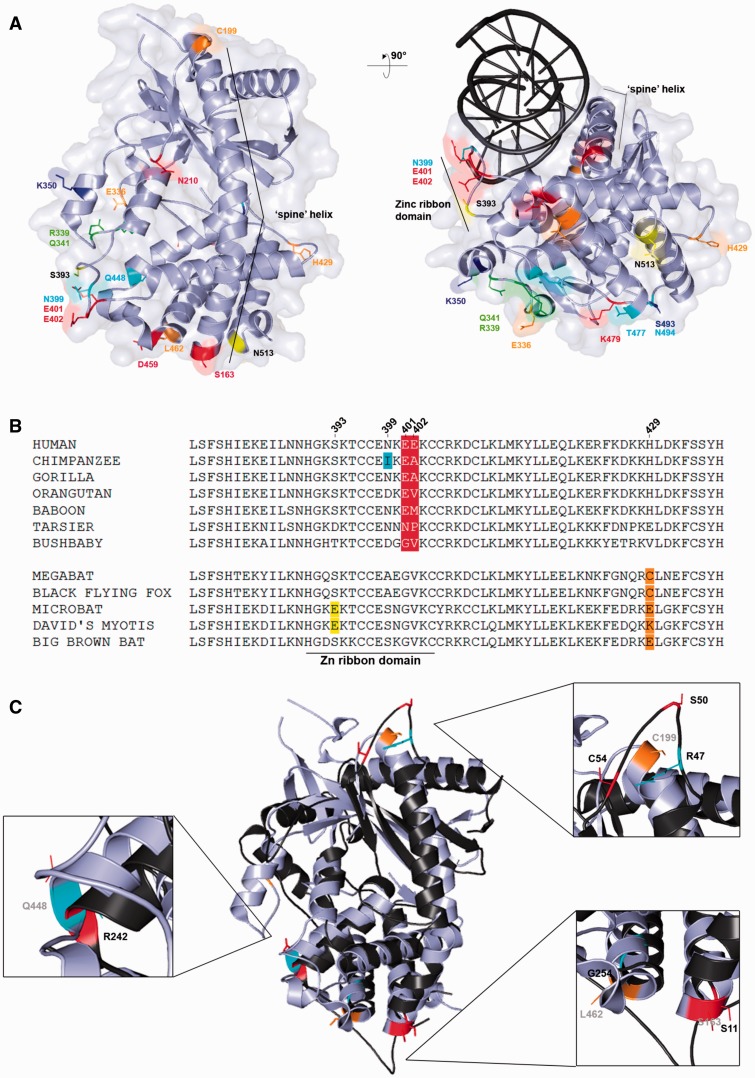
(*A*) Positively selected sites mapped onto the human cGAS structure (PDB code: 4O67).Color codes are as follows: Red, positively selected sites in the primate phylogeny; orange, positively selected sites in the bat phylogeny; yellow, lineage-specific sites; cyan, positively selected sites in the chimpanzee lineage; blue, positively selected sites in the gorilla lineage; green, positively selected sites in more than one lineage among human, chimpanzee, and gorilla. The cGAS–dsDNA complex was obtained by superimposing the human cGAS structure (PDB code: 4O67) with the porcine cGAS–dsDNA complex. The porcine cGAS structure is omitted. (*B*) Multiple alignment of cGAS amino acids 377–437 (a portion of the sequence encompassing the zinc ribbon domain) for a few of representative primates and bats species. (*C*) Superimposition of the structure of the cGAS (PDB code: 4O67, light gray) and OAS1 (PDB code: 4IG8, black). Enlargements highlight positively selected sites located in the corresponding regions of the two different enzymes. Color codes are as in (*A*).

As mentioned above, OAS1 shares striking functional and structural similarities with cGAS; a distinctive feature of this latter is the presence of a Zinc-ribbon domain that is important for double-stranded DNA (dsDNA) binding and DNA-induced dimerization. We found three positively selected sites in primates (I399, E401, and E402) and one in bats (S393) to be located in this domain (figs. 2 and 5*A* and *B*).

Superimposition of the OAS1 and cGAS 3D structures in complex with the nucleic acid molecule indicated that the S11 residue of OAS1 perfectly matches S163 of cGAS; both residues were positively selected in primates or Homininea and define the N-terminus of the “spine” helix. Interestingly, the C54 site of OAS1 and the C199 residue of cGAS (positively selected in bats) are located at the C-terminus of this same helix (fig. 5*C*). C199 is also in spatial proximity to OAS1 residues S50 and R47. Additional matching residues include L462 (cGAS) and G254 (OAS1), as well as Q448 (cGAS) and R242 (OAS1) (fig. 5*C*).

### Positive Selection Targets Functional Sites in TMEM173 and RNASEL

In *TMEM173*, we found six positively selected sites in bats; these sites mainly localize in functional regions of the protein. L23, S65, and V113 are in the transmembrane regions of the receptor, which are important for protein dimerization (Sun et al. 2009). T181 immediately flanks the second ER retention signal in the protein sequence (fig. 2). Interestingly, we also found R78, that is part of the first RXR retention minimal motif (Sun et al. 2009), as positively selected in the chimpanzee lineage. Other primates display amino acids different from arginine at this position, suggesting variable localization of STING in distinct species (supplementary fig. S2, Supplementary Material online).

**Fig. 6.—:**
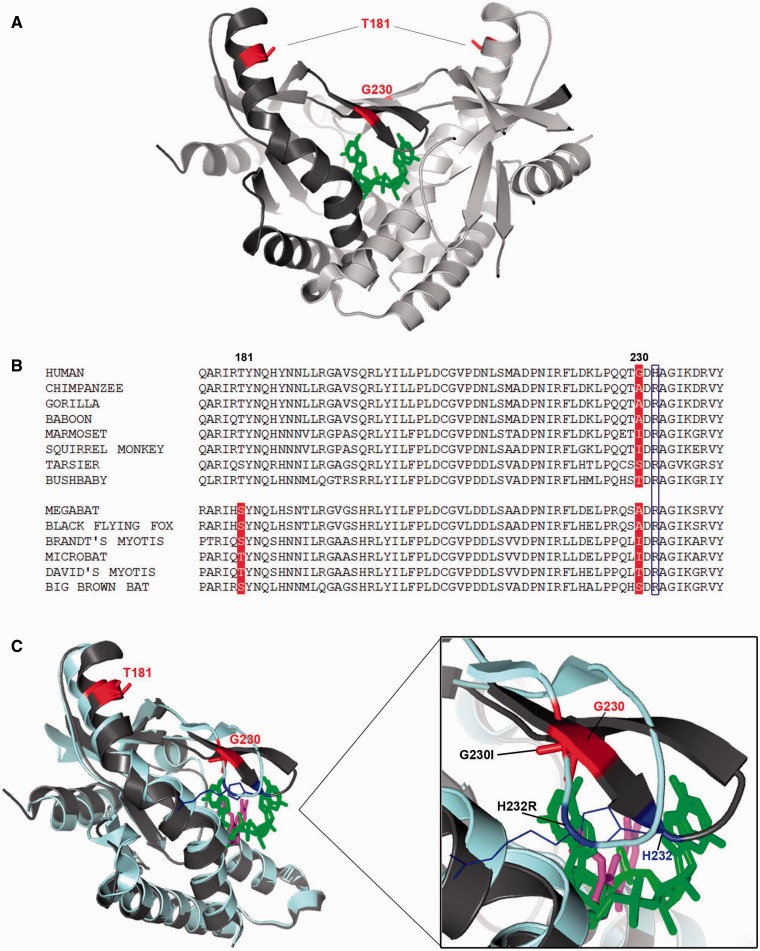
(*A*) Positively selected sites mapped onto the human STING dimeric structure in complex with [G(2′,5′)pA(3′,5′)p] (green) (PDB code: 4LOH). The two monomers are colored in dark and light gray. Positively selected sites in both orders are in red. (*B*) Multiple alignment of cGAS amino acids 176–240 for a few of representative primate and bat species. Positively selected in primates and/or bats are in red; position 232 is boxed in blue. (*C*) Superimposition of the structure of the wt STING monomer (dark gray) in complex with [G(2′,5′)pA(3′,5′)p]) (green) (PDB code: 4LOH) and the STING double mutant (G230I, H232R) (pale cyan) in complex with DMXAA (magenta) (PDB code: 4QXP). The different conformation of the loop covering the dNTPs binding site is enlarged. [G(2′,5′)pA(3′,5′)p] and DMXAA are represented sticks.

Position 230 was identified as positively selected both in the primate and in the bat phylogenies. This site is also polymorphic in human populations (G230A, rs78233829). Residue 230 is located in a loop forming the lid region that clamps onto the cyclic dinucleotide binding pocket of the receptor; mutations of this residue affect the conformation of the protein C-terminal domain and also the binding to cyclic dinucleotides, as well as to pharmacological mimetic drugs (Gao, Ascano, Zillinger, et al. 2013; Yi et al. 2013; Gao et al. 2014) (fig. 6).

Finally, position 146 (positively selected in bats) immediately flanks a residue (V147, human sequence) that was recently shown to determine the constitutive activation of STING, irrespective of cGAMP stimulation, when mutated to leucine in humans (Liu et al. 2014).

**Fig. 7.—:**
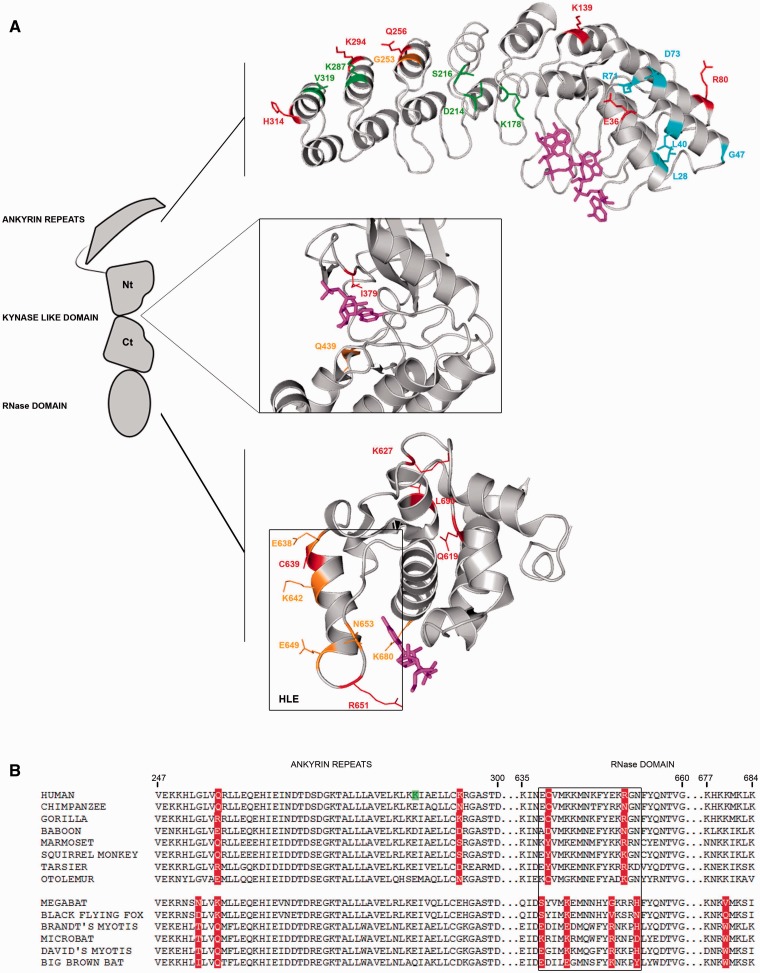
(*A*) Positively selected sites mapped onto the human RNase L structure (PDB code: 4OAV). Enlargements show the ankyrin repeats in complex with 2–5pppA_7_ (magenta), the ATP binding site of the protein kinase-like domain in complex with AMP-PCP (magenta), and the RNase domain in complex with RNA (two sugar-phosphate groups and one pyrimidine nucleobase solved, magenta). Color codes are as follows: Red, positively selected sites in the primate phylogeny; orange, positively selected sites in the bat phylogeny; cyan, positively selected sites in the chimpanzee lineage; green, positively selected sites in the human lineage. The HLE is boxed in black. (*B*) Multiple alignment of RNase L amino acids 247–684 (a portion of the sequence encompassing the HLE, black boxed) for a few of representative primates and bats species. Positively selected sites in primates and bats are in red, the positive selected site in the human lineage is in green.

The evolutionary history of *RNASEL* in Primates had previously been analyzed (Jin et al. 2012). Herein we confirmed most sites reported by Jin et al. (2012) and detected few more sites, possibly as a result of increased species number. Most positively selected sites detected by gammaMap or BEB/MEME localize to the ankyrin domain, whereas residues 379 and 439 (positively selected in primates and bats, respectively) lie in the ATP binding pocket of the kinase-like domain (fig. 7*A*). Although this domain lacks the phosphotransfer activity, nucleotide binding is maintained and required for the assembly of a functional RNase dimer (Huang et al. 2014). In bats we also found a positively selected site at position 680, within a positively charged residue patch (_677_KHKKMKLK_684_, human sequence) that possibly interacts with the acidic ankyrin domain (Tanaka et al. 2004) (fig. 7*B*). The interaction is thought to inhibit RNase L activity in absence of 2′–5′ poliadenylates. In Chiroptera, this position is mainly occupied by hydrophobic residues such as valine and tryptophan (fig. 7*B*). Finally, positively selected sites were detected in the RNase domain: Most of these (E638, C639, K642, E649, R651, and N653) are part of a α-helix/loop element (HLE) that creates the substrate-binding pockets (fig. 7*B*). Deletions in the HLE modulate the substrate preference of human RNase L (Korennykh et al. 2009; Han et al. 2014).

## Discussion

Infections account for about 66% and 72% of deaths among wild chimpanzee and extant human traditional societies, respectively (Finch 2010); these figures underscore the relevance of infectious agents as powerful selective forces during the evolutionary history of Primates and, most likely, of other mammals. In fact, genetic data revealed that, among environmental factors, pathogens represented the strongest selective pressure for humans (Fumagalli et al. 2011) and several reports used inter- or intraspecies diversity data to describe widespread adaptive evolution at immune response loci (Barreiro and Quintana-Murci 2010; Daugherty and Malik 2012; Quintana-Murci and Clark 2013). Specific selective events act to increase the host resistance against one or more pathogens and ample evidence indicates that the selective pressure exerted by past infections contributed to shaping the susceptibility to present-day pathogens (Kaiser et al. 2007; Emerman and Malik 2010). Also, it has previously been suggested that, by generating diversity, selection may induce species-specific differences in the response to pharmacological compounds (e.g., vaccine adjuvants), suggesting caution when extrapolating results obtained in model organisms (Werling et al. 2009; Forni et al. 2013). For these reasons, evolutionary analyses of immune response genes may provide valuable information on the molecular determinants underlying species-specific infection susceptibility and may clarify the differential response to natural or synthetic molecules.

Herein, we performed evolutionary analysis in primates and bats. These latter were included because of the exceptional wide range of viruses they host without developing evident pathology. Only six bat species are presently available for analysis, possibly resulting in low accuracy and power in positive selection tests (Anisimova et al. 2001). Although we limited the false positive rate by using two different methods to declare a site as positively selected, we may have failed to detect some true positives. Indeed, fewer selected sites were generally detected in Chiroptera than in Primates. Taking this limitation into account, we note that several selected sites were identified in the bat *TMEM173* and *RNASEL* genes, suggesting that selective pressure in these mammals was comparatively stronger for downstream effectors than for PRRs, in accordance with the higher average d*N*/d*S* values (fig. 2).

In line with the tenet that natural selection targets functionally relevant residues, position 230 in *TMEM173* was found to be positively selected in both the primate and bat phylogenies. This site lies in the flexible loop that acts as a lid above the cyclic dinucleotide binding pocket of the receptor (fig. 6). Substitutions at this site greatly affect the response to natural ligands and to mimetic drugs, such as DMXAA (Yi et al. 2013; Gao et al. 2014). In humans, positions 230 and 232 are polymorphic (G230A and H232R). Different alleles at these sites affect STING binding specificity for different substrates, including the canonical 3′–5′ cyclic dinucleotides, known to be synthesized by bacteria, and the noncanonical [G(2′–5′)pA(3′–5′)p] cyclic dinucleotide, that contains a single 2′–5′ phosphodiester bond and is produced by mammalian cGAS (Ablasser et al. 2013; Diner et al. 2013; Gao, Ascano, Wu, et al. 2013; Zhang, Shi, et al. 2013). Although G230 displays a substrate specificity restricted to the noncanonical dinucleotides, the G230A substitution enhances signal transduction at very low concentrations of canonical dinucleotides, because the flexibility of the loop is increased and favors the structural changes that occur upon ligand binding (Yi et al. 2013). Furthermore, even though H232R was demonstrated to be critical for the responsiveness to canonical dinucleotides, the coupled substitution G230A is required to restore a complete enzyme activation on these substrates (Diner and Vance 2014).

Different amino acid residues at position 230 were also shown to be responsible for the species-specific differences in the induction of the type I interferon pathway in response to DMXAA in human and mouse (Gao et al. 2014). Indeed, this mimetic drug showed promising antitumor effects in mice, but failed in human clinical trials because the human protein does not bind to or signal in response to DMXAA (Gao et al. 2014). Functional studies (Gao et al. 2014) nevertheless demonstrated that the substitution of Gly with Ile at position 230 results in the gain of function of human STING for DMXAA recognition.

These observations suggest that binding affinity for natural ligands drove the evolution of the STING binding crevice and eventually resulted in species-specific response to a synthetic compound. In this respect, it is worth noting that the variability of position 230 in primates should be taken into account in the design of DMXAA derivatives for the development of human antitumor and antiviral applications. These same considerations apply to the proposed use of STING-stimulating cyclic nucleotides as vaccine adjuvants (Dubensky et al. 2013).

Adding complexity to STING evolution, we also noted that positive selection in chimpanzee drove the loss of the N-terminal ER-retention signals in STING. Although the C-terminal motif (_178_RIR_180_) was shown to be more important for ER retention, mutagenesis of the N-terminal signal (_78_RYR_80_) resulted in a decreased ER localization of the protein (Sun et al. 2009). Analysis in primates revealed that additional species lack the _78_RXR_80_ signal. In bats, with the exclusion of the big brown bat, which has an intact second motif (_178_RIR_180_), no RXR motif is present in STING, nor is any of the other two motifs (KKXX and H/KDEL) usually associated with ER localization (Sun et al. 2009). The mitochondrial localization of human STING initially reported by Zhong et al. (2009) has remained controversial (Ishikawa and Barber 2008; Burdette and Vance 2013). Recently, it has been suggested that the protein localizes to mitochondria-associated membranes, where its interaction with MAVS and RIG-I occurs (Scott 2009; Horner et al. 2011). It will be interesting to assess whether species-specific differences in TMEM173 localization exist and how these affect immune response and interaction with viral-encoded inhibitors (Burdette and Vance 2013).

Similarly to TMEM173, positively selected sites were identified in functional domains of RNase L. Several positively selected sites (E638, K642, E649, and N653 in Chiroptera; C639 and R651 in Primates) localize to the short HLE element, which constitutes the binding-pocket for RNA and modulates the preference of the enzyme for single-stranded RNA molecules or stem loops (Korennykh et al. 2009; Han et al. 2014) (fig. 7). Experiments in cell lines indicated that human RNase L cleaves HCV RNA predominately at UA and UU dinucleotides within loops of predicted stem–loop structures (Han and Barton 2002). More recently, a phylogenetically conserved RNA structure in the open reading frame of poliovirus (and other group C enteroviruses) was found to function as a competitive inhibitor of RNase L (Han et al. 2007). Specific stem loops motifs were found to be important for the inhibitory activity and to account for unusual resistance of poliovirus to RNase L-mediated cleavage (Townsend et al. 2008). Thus, positively selected sites in the HLE represent excellent candidates as modulators of RNase L cleavage rate or susceptibility to inhibitors.

The RNase L ankyrin repeats domain was strongly targeted by selection, as well. Most selected sites lie in the loop between the two antiparallel alpha-helices and in the outer helix of the ankyrin module; even though none is directly involved in the 2′–5′ oligoadenylates binding, they could potentially mediate the dimerization process or the interaction with other proteins. Intriguingly, the ankyrin domain of murine RNase L is the molecular target of L*, a protein of Theiler*’*s Virus, a neurotropic picornavirus. The interaction between RNase L and L* is strictly species-specific: The viral protein is unable to inhibit RNase L of nonmurine origin (Sorgeloos et al. 2013). Although our analysis did not include rodents, these observations indicate that the ankyrin repeat domain may be a target of virus-encoded inhibitors.

Overall, these data suggest that the selective pressure acting on STING and RNase L is mainly related to the modulation of molecular recognition and, possibly, to the escape from viral inhibitors. It will be interesting to evaluate whether the positively selected sites we detected in bats contribute to the exceptional adaptation of these mammals to different viral pathogens.

Analysis of the PRRs underscored major signatures of adaptive evolution for cGAS in both Chiroptera and Primates, whereas OAS1 was strongly targeted by selection in Primates, and much more weakly in bats. We found several positively selected sites to be located in the relatively short cGAS-specific zinc ribbon domain. This structure is thought to act as a molecular ruler and to endow cGAS with the ability of binding the B-form but not A-form of nucleic acids, which is instead recognized by OAS1 (Civril et al. 2013; Gao, Ascano, Wu, et al. 2013; Kranzusch et al. 2013). Thus, positive selection in this domain may act to hone in the function of this protruding loop, which is responsible both for nucleic acid recognition and for protein dimerization, two essential steps for full enzyme activation (Li et al. 2013; Zhang et al. 2014).

Human cGAS and OAS1 recognize nucleic acids through sequence-independent interactions to the minor groove, mainly mediated by a positively charged platform on the protein surface (Hornung et al. 2014). A long alpha helix, called “spine” helix, opposite to the active site crevice, is the major structural component of the platform (Hornung et al. 2014). For an efficient activation in vitro, human OAS1 and cGAS require dsRNA molecules greater than 17 bp long (Donovan et al. 2013) and dsDNA molecules greater than 20 bp (Kranzusch et al. 2013), respectively. Intriguingly, we found positively selected sites at the double ends of the spine helix of both proteins, as shown by the superimposition of the 3D structures. As cGAS and OAS1 use double-stranded acid topology to distinguish between DNA and RNA and for specific self-activation upon binding, domains directly involved in nucleic acid recognition may have evolved adaptively to respond to specific PAMPs or to optimize enzyme activation. A similar evolutionary scenario has been recently proposed for positively selected sites in the pincer region of RIG-I, another PRR (Lemos de Matos et al. 2013; Cagliani, Forni, Tresoldi, et al. 2014; Rawling et al. 2015). Thus, the selective pressure acting on *OAS* and *MB21D1* genes may be related to PAMP recognition and to the specific mechanism of enzyme activation, which envisages a conformational change. This hypothesis is strengthened by the observation that natural selection often targeted residues located in the same spatial position in different proteins. In this respect, we should add that the duplication events that originated the *OAS* gene family occurred before the radiation of mammalian lineages, although more recent expansions occurred in rodents (Kumar et al. 2000). Consequently, *OAS* family genes are quite divergent in sequence and the possibility that gene conversion between paralogs contributes substantially to their evolution has previously been dismissed (Ferguson et al. 2012). Likewise, gene conversion events between the *OAS1* and *MB21D1* coding regions are extremely unlikely, as the two genes display limited sequence identity, despite the extensive structural and functional similarities. Thus, the several instances of corresponding positions which were targeted by positive selection in *OAS1* and *OAS2*, as well as *OAS1* and *MB21D1*, should be regarded as independent events resulting from selection.

In terms of function and structure, it is worth noting that recent analyses (Kranzusch et al. 2014; Zhu et al. 2014) indicated that cGAS is homologous to bacterial enzymes that synthesize 3′–5′ cGAMP, revealing an evolutionary connection across distinct kingdoms. Based on these structural similarities Kranzusch et al. (2014) showed that single amino acid replacements around the cGAS binding site alter the enzyme*’*s linkage specificity. Although the selected sites in STING may affect the specificity for products with different phosphodiester bonds, we did not detect positively selected sites in or near the cGAS active site, suggesting that the major selective pressure acting on the enzyme was not related to changes in STING ligand specificity. Evolutionary analyses on additional species will be required, though, to address the potential of coevolution for cGAS product specificity and STING binding preferences (Kranzusch et al. 2014).

The combined analysis of intraspecies polymorphism and between-species divergence allows detection of positive selection targets in one species and provides information on the distribution of selective coefficients along the whole gene regions. A previous study of *TLR* gene evolution in humans and great apes revealed a stronger effect of purifying selection in chimpanzees and gorillas compared with humans (Quach et al. 2013). We analyzed these same species and did not detect a similar trend. Nonetheless, in that previous work, the major difference among species was accounted for by TLRs that recognize bacterial PAMPs, whereas the genes we analyzed herein are mainly devoted to antiviral response. In general, the distribution of selection coefficients was relatively similar among the three species, with the exception of *OAS2* and *TMEM173*, which showed a marked preponderance of selectively constrained codons in humans and chimpanzees, respectively.

An interesting observation emerging from the population genetics–phylogenetics analysis is that results were consistent with the long-standing balancing selection scenario that was previously described at the *OAS1* locus in hominids (Ferguson et al. 2012). In fact, we identified shared selected sites in the three species, with some of them polymorphic in chimpanzees, but fixed in humans and gorillas. This observation makes perfect sense if, as shown by Ferguson et al. (2012), two or more haplotypes originated before the split of great apes and were driven to fixation or maintained in the population as a result of selective forces. We also detected fixed positively selected sites shared between gorillas and humans in *MB21D1*, possibly suggesting a similar scenario as in *OAS1*.

Overall, our population genetics–phylogenetics analysis identified several sites which were targeted by positive selection in distinct great ape lineages; these represent extremely promising candidates as modulators of infection susceptibility in these species. Although some of these sites are located in protein regions with clear functional characterization (e.g., at the nucleic acid binding interface, at intracellular trafficking signals), the significance of other selected residues remains elusive. As suggested for the balanced polymorphisms in *OAS1* (Ferguson et al. 2012), selection may act on regions that play a role in protein folding and stability. Also, it will be interesting to investigate whether the diverse evolutionary histories for OAS1 and STING in distinct great ape species resulted from the selective pressure exerted by of one or more pathogens.

## Supplementary Material

Supplementary tables S1–S4 and figures S1 and S2 are available at *Genome Biology and Evolution* online (http://www.gbe.oxfordjournals.org/).

Supplementary Data
