# Supplementary material for: OASes and STING: Adaptive Evolution in Concert
Source: Genome Biol Evol. 2015 Mar 9;7(4):1016–32. doi: 10.1093/gbe/evv046 (PMC4419793; doi:10.1093/gbe/evv046)
Supplement: Supplementary Data [file supp_7_4_1016__index.html]

OASes and STING: Adaptive Evolution in Concert — Supplementary Data 

# OASes and STING: Adaptive Evolution in Concert

## Supplementary Data

files

**Files in this Data Supplement:**

- Supplementary Data - pdf file
