# Supplementary material for: OASes and STING: Adaptive Evolution in Concert
Source: Genome Biol Evol. 2015 Mar 9;7(4):1016–32. doi: 10.1093/gbe/evv046 (PMC4419793; doi:10.1093/gbe/evv046)
Supplement: Supplementary Data [file supp_evv046_Supplementary_DATA.pdf]

SUPPLEMENTARY DATA

Supplementary Figure S1. Branch-site analysis of positive selection for *OAS1* and *RNASEL* genes. BS-REL analysis for *OAS1* (Chiroptera, A) and *RNASEL* (Primates, B) genes. Branch lengths are scaled to the expected number of substitutions per nucleotide, and branch colors indicate the strength of selection (dN/dS or ω). Red, positive selection (ω>5); blue, purifying selection (ω = 0); gray, neutral evolution (ω = 1). The proportion of each color represents the fraction of the sequence undergoing the corresponding class of selection. Thick branches indicate statistical support for evolution under episodic diversifying selection as determined by BS-REL. Red dots denote the branch that was confirmed to be under positive selection using the codeml branch-site models.

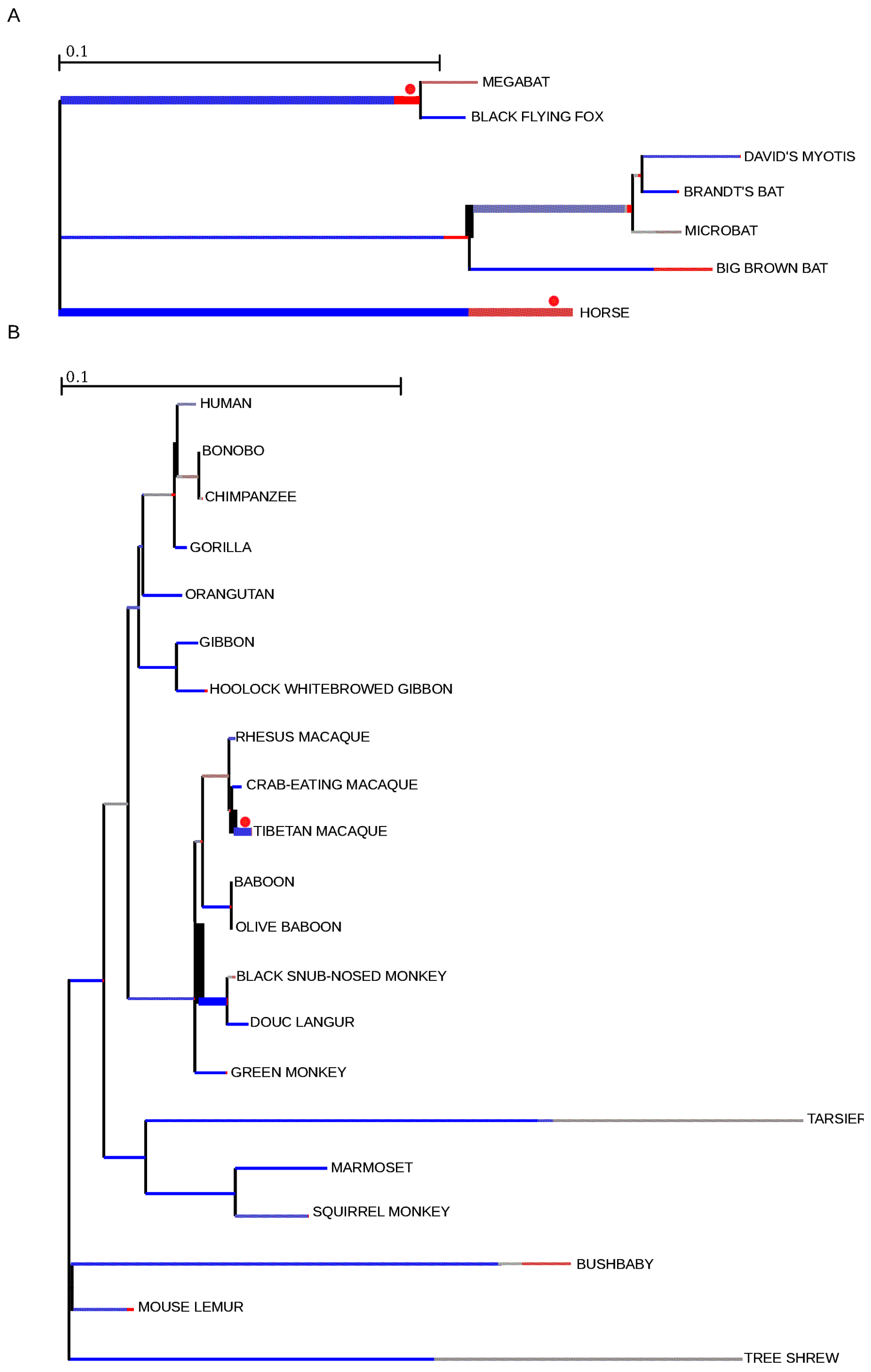

Supplementary Figure S2. Multiple alignment of the STING N-terminus.
The two ER retention minimal motifs are boxed in blue. Positively selected sites in the bat phylogeny are in red; position 78, which is a positively selected site in chimpanzee lineage, is in cyan.

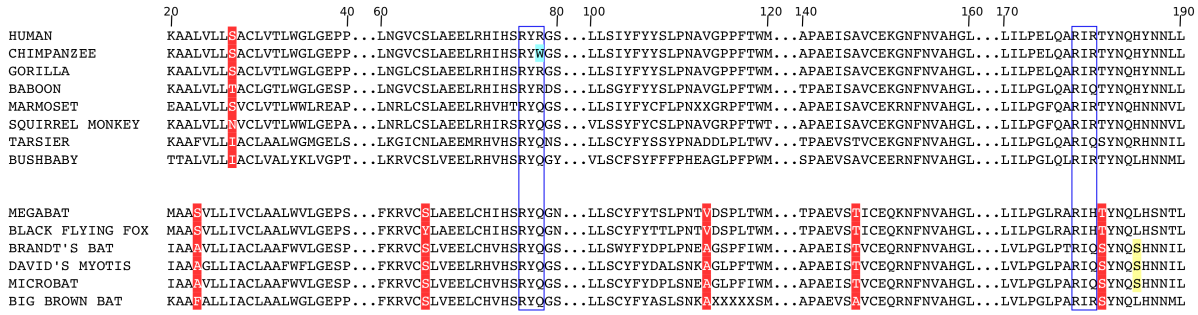

Supplementary Table 1. Genomic position for *OAS* genes, *MB21D1*, *RNASEL*, and *TMEM173*.

Gene	Alias	Genomic location	Size (aa)
OAS1	*OIAS*	Chr12:113,344,739 - 113,357,712	400
OAS2	-	Chr12:113,416,274 - 113,449,528	719
OAS3	*P/OKcl.4*	Chr12:113,376,249 - 113,411,054	1087
OASL	*TRIP14*	Chr12:121,458,095 -121,477,045	514
MB21D1	*CGAS, C6orf150*	Chr6:74,134,856 - 74,162,043	522
RNASEL	*RNS4*	Chr1:182,542,769 - 182,558,394	741
TMEM173	*ERIS, MITA, STING*	Chr5:138,855,113 - 138,862,375	379

Supplementary Table 2. List of species.

Common name	Scientific name

**Primates**	
Baboon	*Papio hamadryas*
Black snub-nosed monkey	*Pygathrix bieti*
Bonobo	*Pan paniscus*
Bushbaby	*Otolemur garnettii*
Chimpanzee	*Pan troglodytes*
Crab-eating macaque	*Macaca fascicularis*
Douc langur	*Pygathrix nemaeus*
Gibbon	*Nomascus leucogenys*
Gorilla	*Gorilla gorilla gorilla*
Green monkey	*Chlorocebus sabaeus*
Hoolock whitebrowed gibbon	*Bunopithecus hoolock*
Human	*Homo sapiens*
Macaque	*Macaca mulatta*
Marmoset	*Callithrix jacchus*
Mouse lemur	*Microcebus murinus*
Olive baboon	*Papio anubis*
Squirrel monkey	*Saimiri boliviensis*
Sumatra orangutan	*Pongo abelii*
Tarsier	*Tarsius syrichta*
Tibetan macaque	*Macaca thibetana*
(Tree shrew)*	*(Tupaia belangeri)*
**Chiroptera**	
Big brown bat	*Eptesicus fuscus*
Black flying fox	*Pteropus alecto*
Brandt's bat	*Myotis brandtii*
David's myotis	*Myotis davidii*
Megabat	*Pteropus vampyrus*
Microbat	*Myotis lucifugus*
(Horse)*	*(Equus caballus)*

()* Species used as outgroup

Supplementary Table 3. Likelihood ratio test statistics for models of variable selective pressure among sites (codon frequency model: F61).

Gene	N species	-2ΔLnL	*p* value	% of sites (average dN/dS)
OAS1				
M1a vs M2a				
Primates	17	74.50	6.64×10^{-17}	21.3% (3.1)
Chiroptera	7	32.24	9.97×10^{-8}	11.1% (4.3)
M7 vs M8				
Primates	17	76.98	1.92×10^{-17}	24.0% (2.9)
Chiroptera	7	32.73	7.80×10^{-8}	12.1% (4.0)
OAS2				
M1a vs M2a				
Primates	16	64.48	9.98×10^{-15}	9.5% (3.0)
M7 vs M8				
Primates	16	89.91	2.99×10^{-20}	14.5% (2.5)
MB21D1				
M1a vs M2a				
Primates	16	43.44	3.69×10^{-10}	8.1% (3.5)
Chiroptera	6	12.12	0.002	40.3% (1.9)
M7 vs M8				
Primates	16	45.89	1.08×10^{-10}	11.4% (2.9)
Chiroptera	6	13.08	0.001	40.2% (1.9)
RNASEL				
M1a vs M2a				
Primates	21	38.97	3.44×10^{-9}	5.9% (3.1)
Chiroptera	7	38.04	5.48×10^{-9}	9.2% (3.8)
M7 vs M8				
Primates	21	50.95	8.64×10^{-12}	8.8% (2.6)
Chiroptera	7	37.97	5.68×10^{-9}	10.4% (3.6)
TMEM173				
M1a vs M2a				
Primates	17	6.01	0.05	30.1% (1.3)
Chiroptera	7	24.13	5.75×10^{-6}	24.4% (3.0)
M7 vs M8				
Primates	17	6.63	0.04	29.8% (1.3)
Chiroptera	7	37.97	5.68×10^{-9}	10.4% (3.6)

Note: M1a is a nearly neutral model that assumes one ω class between 0 and 1. and one class with ω=1; M2a (positive selection model) is the same as M1a plus an extra class of ω >1; M7 (null model) assumes that 0<ω<1 is beta distributed among sites in 10 classes; M8 (selection model) has an extra class with ω >=1; 2ΔLnL: twice the difference of the natural logs of the maximum likelihood of the models being compared; *p* value: *p* value of rejecting the neutral models (M1a or M7) in favor of the positive selection model (M2a or M8); % of sites (average dN/dS): estimated percentage of sites evolving under positive selection by M8 and M2a (dN/dS for these codons).

Supplementary Table 4. Likelihood ratio test statistics for models of variable selective pressure along branches and branch-site tests in Primates and Chiroptera.

PRIMATES					
**Gene**	**Model**	**-2ΔLnL**	**Degrees of freedom**	***p* value**	
OAS1					
	**M0** vs **M1**				
	F3X4	69.92	31	7.8x10-5	
	F61	68.21	31	0.0001	
MB21D1					
	**M0** vs **M1**				
	F3X4	56.56	29	0.002	
	F61	56.70	29	0.002	
RNASEL					
	**M0** vs **M1**				
	F3X4	72.08	39	0.001	
	F61	71.17	39	0.001	
**Gene**	**Foreground branch (MA vs MA1)**	**-2ΔLnL**	**Degrees of freedom**	***p* value**	**Sites**
OAS1					
	Chimpanzee	2.95	1	0.09 (0.17)	-
	Platyrrhini	0	1	1 (1)	-
MB21D1					
	Hominidea	6.07	1	0.01 (0.01)	S163
	Homininae	11.03	1	0.001 (0.002)	T130, N210, E402
	Orangutan	10.12	1	0.001 (0.002)	-
RNASEL					
	Tibetan macaque	13.02	1	0.0003 (0.0006)	-
	Presbytini	0	1	1 (1)	-
**CHIROPTERA**					
**Gene**	**Model**	**-2ΔLnL**	**Degrees of freedom**	***p* value**	
OAS1					
	**M0** vs **M1**				
	F3X4	26.72	11	0.005	
	F61	24.10	11	0.01	
MB21D1					
	**M0** vs **M1**				
	F3X4	29.16	9	0.0006	
	F61	30.39	9	0.0003	
**Gene**	**Foreground branch (MA vs MA1)**	**-2ΔLnL**	**Degrees of freedom**	***p* value**	**Sites**
OAS1					
	Horse	9.56	1	0.002 (0.01)	-
	Pteropodidae	4.83	1	0.03 (0.04)	-
	Myotinae	0.44	1	0.51 (0.51)	-
MB21D1					
	Pteropodidae	10.82	1	0.001 (0.001)	H429, N513
	Myotinae	11.57	1	0.0007 (0.001)	S393

Note: M0 and M1 are free-ratio models that assume all branches to have the same ω (M0) or allow each branch to have its own ω (M1).MA and MA1 are branch-site models that assume four classes of sites: the MA model allows a proportion of codons to have ω≥ 1 on the foreground branches, whereas the MA1 model does not. 2ΔLnL is twice the difference of the natural logs of the maximum likelihood of the models being compared.
